# Examining the Mediation Role of Emotion Regulation and Hope in the Impact of COVID‐19 on Psychological Well‐Being and Mental Health

**DOI:** 10.1002/ijop.70216

**Published:** 2026-04-21

**Authors:** Marco Tommasi, Simone Arnò, Aristide Saggino

**Affiliations:** ^1^ Department of Psychology University of Chieti‐Pescara Chieti Italy

**Keywords:** anxiety, cognitive reappraisal, depression, emotional suppression, hope, individual differences, lockdown, mediation analysis, mental health, psychological well‐being

## Abstract

The COVID‐19 pandemic profoundly affected global physical and psychological well‐being. In addition to the loss of lives, lockdowns led to widespread declines in quality of life, particularly in mental health. While some individuals struggled, others showed resilience. This study investigated how changes in life conditions during lockdown—across physical, psychological and social domains—impacted mental health and well‐being. A sample of 184 university students (82.6% female; *M* = 22.8, SD = 4.09) reported perceived improvements or deteriorations in these areas. Their responses were analysed in relation to psychological outcomes. The study also examined the mediating roles of emotion regulation strategies—cognitive reappraisal and expressive suppression—and hope. Although previous research emphasised the protective role of emotion regulation and hope in reducing anxiety and depression, findings from this study revealed that the psychological impact of lockdown was the strongest predictor of mental health and well‐being. Emotion regulation strategies did not significantly mediate these effects. In contrast, hope emerged as the only effective mediator, reducing the negative psychological consequences of lockdown and enhancing resilience and coping. These results underscore the importance of cultivating hope as a central psychological resource to support individuals in managing prolonged adversity and maintaining psychological well‐being.

## 1 Introduction

The COVID‐19 pandemic had a catastrophic impact on global physical and mental health. As of 5 November 2024, over 676 million infections and 6.88 million deaths were reported globally (source: https://coronavirus.jhu.edu/map.html) The virus disrupted health trends, with surges in cases and mortality, particularly during late 2020. It ranked as a leading cause of death, severely affecting older adults, while younger populations faced increased mortality from drug overdoses, accidents and suicide (Woolf et al. 2020). The pandemic also stagnated life expectancy and highlighted the need for coordinated public health measures and federal planning (Koh et al. 2021).

Beyond physical health, the pandemic profoundly impacted psychological well‐being. Isolation, lockdowns, and uncertainty heightened anxiety and depression worldwide. Italian students reported significant psychological distress due to isolation (Villani et al. 2021), while U.K. data revealed increased clinically significant distress during lockdowns (Gray et al. 2020). German university students experienced elevated depressive symptoms, loneliness and stress (Kohls et al. 2021).

Healthcare workers faced unique challenges, including fear of infection, insufficient protective equipment and moral injury from ethical conflicts, contributing to adverse mental health outcomes (De Kock et al. 2021). Economic hardships further exacerbated anxiety, PTSD, and identity crises (Godinić and Obrenovic 2020), with physical symptoms like sleep disorders and exhaustion commonly reported (Tommasi et al. 2020).

Resilience and coping strategies, such as emotional reappraisal, hope and positive habits like exercise and social support, mitigated mental health impacts (Killgore et al. 2020). Individuals with higher resilience were better equipped to manage stress and adapt to disruptions, demonstrating the critical role of emotion regulation and hope in fostering psychological well‐being.

In this context, the present study focuses on two key psychological constructs—emotion regulation and hope—that may mediate the impact of lockdown‐related stressors on mental health. Emotion regulation refers to the processes by which individuals influence their emotions, how they experience and express them. Two widely studied strategies are cognitive reappraisal and expressive suppression. Cognitive reappraisal involves reframing a situation to alter its emotional impact and is generally associated with adaptive outcomes such as reduced anxiety and depression. Expressive suppression, by contrast, involves inhibiting emotional expression and is often linked to negative outcomes like increased distress and reduced social functioning.

Cognitive reappraisal (CR) is an adaptive emotion regulation strategy that involves changing one's perspective on a situation to alter its emotional impact. For example, reframing negative feedback as an opportunity for growth can transform frustration into motivation, enhancing emotional well‐being and fostering personal development. CR is associated with lower levels of anxiety and depression, improved well‐being, and increased positive affect, emotional coping and flourishing (Peh et al. 2017; Vally and Ahmed 2020). It acts as a buffer against negative outcomes, particularly for individuals with low hope (Peh et al. 2017).

Expressive suppression (ES), on the other hand, involves inhibiting outward emotional expression. Unlike CR, ES is often linked to negative psychological outcomes such as heightened anxiety, depression, and reduced social functioning. It limits emotional and social coping and is associated with greater distress in contexts of social anxiety and depression (Dryman and Heimberg 2018). ES does not contribute to well‐being for those with low hope and may increase stress and emotional burden (Peh et al. 2017).

Hope is an optimistic outlook characterised by agency (confidence in achieving goals) and pathways (envisioning routes to goals). Hope is conceptualised here as a dispositional trait comprising agency (goal‐directed energy) and pathways (planning to meet goals). Hope acts as a protective factor against anxiety and depression, promoting resilience and positive mental health (Richards 2002). It enhances life satisfaction, resilience and psychological well‐being, mediating the effect of CR on anxiety and depression. Hope also fosters post‐traumatic growth and is a predictor of positive recovery outcomes in depression treatment (Leite et al. 2019). During the pandemic lockdown, hope played a crucial role in helping individuals cope with uncertainty and disruption (Tommasi et al. 2020).

CR serves as a mechanism through which factors like social support and optimism influence well‐being. It mediates relationships between optimism, life satisfaction and depression, as well as between social participation and post‐traumatic growth (Laslo‐Roth et al. 2022). Conversely, ES is associated with increased stress and reduced life satisfaction, as it prevents the healthy processing of emotions (Peh et al. 2017). While ES may play a role in emotion regulation, it does not promote psychological health. ES appears to be a less adaptive mediator in relation to well‐being. For example, Graça and Brandão found that difficulties in emotion regulation, rather than specific strategies like suppression, explained the negative impact of certain types of religious/spiritual coping on well‐being (Graça and Brandão 2024). This suggests that while suppression might play a role in emotional regulation, it may not mediate relationships in ways that promote psychological health.

Hope is a significant mediator in fostering resilience and positive growth, particularly during challenging times. For instance, hope mediated the relationship between social participation and post‐traumatic growth during the COVID‐19 pandemic, highlighting its role in enhancing coping and adaptation (Laslo‐Roth et al. 2022). Similarly, Akfirat found that hope, along with other psychological resources, predicted well‐being in pre‐service teachers, emphasising its importance in building resilience (Akfirat 2020).

Research consistently demonstrates that hope and CR serve as adaptive mediators, promoting resilience, well‐being and growth. In contrast, ES is associated with less adaptive outcomes and generally lacks a significant positive mediating effect. Mediation analysis clarifies the role of intermediaries, such as CR and hope, in the relationship between stressors and psychological well‐being. This method helps identify if changes in these mediators can reduce the negative impact of life events. This framework leads to the following research questions: (1) To what extent do emotion regulation strategies and hope mediate the relationship between perceived lockdown impact and psychological distress? (2) Are CR and hope associated with better mental health outcomes compared to ES? These questions are addressed through a mediation model tested on a sample of university students who experienced the COVID‐19 lockdown. Throughout the manuscript, the term ‘lockdown impact’ consistently refers to perceived changes from pre‐lockdown baseline levels to the end of the lockdown period, rather than comparisons made during intermediate stages.

Our study investigates whether CR, ES and hope mediate the negative effects of the COVID‐19 pandemic on psychological well‐being and mental health. If confirmed, interventions targeting these mediators could mitigate the pandemic's psychological toll. For example, cognitive behavioural therapy (CBT) can develop CR skills, reducing anxiety and enhancing emotional regulation. Studies show that CBT not only decreases negative emotions but also increases brain activity related to reappraisal, indicating neurobiological changes (Goldin et al. 2013).

Reducing reliance on ES and promoting alternative approaches, such as constructive emotional expression and mindfulness, can also improve emotional well‐being. Hope, as a psychological resource, can be strengthened through goal‐setting, identifying pathways and building a sense of agency. Structured interventions targeting hope, such as motivational exercises, enhance both psychological and physical well‐being (Chan et al. 2019). Hope also helps the reinterpretation of challenges positively, improving job satisfaction and fostering healthier behaviours (Reis and Hoppe 2015).

In our analysis, we tested whether CR, ES and hope mediated the pandemic's impact on well‐being by comparing direct and indirect effects. Our primary aim was to elucidate the mechanisms underlying the relationship between lockdown experiences and psychological adjustment. A mediation model allows us to examine how these experiences influence outcomes, whereas a moderation model would address when or for whom these effects occur. With this model, we explored whether the experience of living through such a situation, beyond its direct effects on mental health and well‐being, could also have indirect effects mediated by personal resources such as resilience and emotional regulation strategies, which may amplify or mitigate the impact of the experience itself.

Our hypotheses were:

*Negative physical, psychological and social impact of lockdown reduces the level of psychological well‐being and mental health*.

*CR can counteract and reduce the lockdown negative impacts on psychological well‐being and mental health*.

*ES is expected to play a less significant role on psychological well‐being and mental health in relation to CR*.

*Hope was hypothesised to be a crucial factor in amplifying resilience and to reduce negative lockdown effects on psychological well‐being and mental health*.

Prior empirical research has demonstrated that hope and resilience often mediate the relationship between stressful life events and psychological well‐being (Laslo‐Roth et al. 2022; Yıldırım et al. 2020).

## 2 Methods

### 2.1 Participants

184 university students (82.6% females) participated in this study. Mean age was 22.8 years (SD = 4.09). 64.7% of participants were involved in stable affective relationships (e.g., engagement, marriage); 70.7% of participants declared having moderate or high satisfactory human relationships and 44.0% declared they did not play sports at all after lockdown.

### 2.2 Materials

We used self‐report tests to measure the negative or positive lockdown effects on life, emotion regulation (CR and ES), hope, mental health and psychological well‐being in participants. We also used a scale to measure social desirability.

### 2.3 Lockdown Effects

The lockdown positive or negative impact on everyday life was measured asking participants how much their life conditions changed (positively or negatively) from the beginning to the end of the lockdown period. All items assessing lockdown impact referred to perceived changes from the period immediately before the national lockdown to the end of the lockdown period. A Likert scale from 1 to 5 was used, with 1 indicating that *life conditions highly worsened* and 5 indicating that *they highly improved after the lockdown*. The scale consisted of three items assessing the impact of lockdown on three principal areas: physical (‘During the COVID‐19 lockdown period, your physical health compared to before highly worsened/improved’), psychological (‘During the COVID‐19 lockdown period, your psychological and mental health compared to before highly worsened/improved’) and social life area (‘During the COVID‐19 lockdown period, your relationships with friends and family compared to before highly worsened/improved’). Given that all three items assess perceived changes in personal well‐being during the COVID‐19 lockdown, moderate positive inter‐correlations are expected. A decline (or improvement) in one domain—such as psychological health—is likely to be connected with similar trends in physical health and social relationships due to the interconnected nature of these experiences. However, the strength of these correlations may vary depending on individual differences and contextual factors, such as coping strategies or social support. Assessing these inter‐correlations helps to understand whether the perceived impact of the lockdown was generalised across life domains or more domain‐specific.

### 2.4 Emotion Regulation

We used the Emotion Regulation Questionnaire with 10 items. The scale consists of two subscales, one for assessing CR (e.g., ‘When I'm faced with a stressful situation, I make myself think about it in a way that helps me stay calm’) and the other for assessing ES (e.g., ‘I keep my emotions to myself’). Item answers are on a 7‐point Likert scale from 1 (*completely disagree*) to 7 (*completely agree*). Higher the score, higher individual reappraisal or suppression. We used the Italian version of the scale (Balzarotti et al. 2010).

### 2.5 Hope

We used two scales to assess the level of hope in participants, the Snyder Hope Scale and the Hope Herth Index. The Snyder Hope Scale consists of 12 items (Snyder et al. 1991). Some items composed the pathways subscale and the agency subscale. According to the authors, Pathways refer to a sense or belief to be able to generate successful plans to reach goals (e.g., ‘I can think of many ways to get out of a jam’), while agency (e.g., ‘I energetically pursue my goals’) refers to a sense of successful determination in meeting goals in the past, present and future. Hope is defined as a cognitive set based on sense of successful agency (goal‐directed determination) and pathways (planning of way to reach goals). Item answers are on a 4‐point Likert scale from 1 (*definitely false*) to 4 (*definitely true*). Higher the score, higher the pathways or agency component of hope. The Hope Herth Index (Herth 1992) consists of 12 items and according to authors the scale measure three principal components of hope: (a) temporality and future that measures the cognitive component of hope, in particular the belief that there are goals with positive outcomes (e.g., ‘I have a positive outlook towards life’); (b) readiness and expectancy that measures the affective‐behavioural dimension of hope, in particular positive feelings, emotions and a sense of optimistic directionality in life (e.g., ‘I have a sense of direction’); (c) interconnectedness that measures the social component of hope, in particular a sense to be part of something, to receive comfort and to be able to give an receive love (e.g., ‘I have a faith that gives me comfort’). Item answers are on 4‐point Likert scale from 1 (*strongly disagree*) to 4 (*strongly agree*). Higher the score, higher the cognitive, affective and social component of hope.

### 2.6 Mental Health

Mental health was measured with the Brief Symptom Inventory‐18 items (Asner‐Self et al. 2006). Six items measures somatisation or the intensity of psychosomatic symptoms (e.g., ‘Pains in heart or chest’), six items measures depression (e.g., ‘Thoughts of ending your life’) and six items measure anxiety (e.g., ‘Nervousness or shakiness inside’). Items answers are on a 5‐point Likert scale from 0 (*not at all*) to 4 (*extremely*). High scores indicate high level of psychological impairment and low mental health.

### 2.7 Psychological Well‐Being

Psychological well‐being was assessed with Subjective Happiness Scale (Lyubomirsky and Lepper 1999) and Basic Psychological Needs Scale (La Guardia et al. 2000). The Subjective Happiness Scale is a four item scale (e.g., ‘In general, I consider myself not a very happy/a very happy person’) with item answers on a 7‐point Likert scale from 1 (*not at all or not very happy*) to 7 (*completely or very happy*). This scale measures the hedonic component of psychological well‐being. Higher the score, higher the subjective happiness. The Basic Psychological Needs Scale measures the eudaimonic component of psychological well‐being. The scale consists of 21 items with item answers on a 5‐point Likert scale from 1 (*never true*) to 5 (*completely true*) and it is articulated into three subscales that are: (a) autonomy which assesses the need of independence and self‐regulation in individuals (e.g., ‘I feel like I am free to decide for myself how to live my life’); (b) competence which assesses the need to feel competent and able to deal with difficult task or situations (e.g., ‘Often, I do not feel very competent’); (c) Relatedness which assesses the need to develop intimate and significant relationships with others (e.g., ‘I really like the people I interact with’). Higher the score, higher the individual autonomy, competence and relatedness.

### 2.8 Social Desirability

Social desirability was assessed with the Marlowe‐Crowne scale (Crowne and Marlowe 1960). The scale consists of nine items (e.g., ‘I am always courteous, even to people who are disagreeable’) on a 5‐point Likert scale form 1 (*false*) to 5 (*true*). Higher the score higher the social desirability. A positive correlation with MC indicates a tendency to overestimate the psychological characteristics, tendency or trait, while a negative correlation indicates a tendency to underestimate.

### 2.9 Procedure

Participants were contacted through advertisements posted on websites or peer‐to‐peer contacts. Privacy of participants was guaranteed according to the Italian and European law (Italian law no. 196/2003 and EU GDPR 679/2016, respectively). This study followed the Declaration of Helsinki ethical principles for research involving human subjects and was approved by the Institutional Review Board of Psychology of the Department of Psychology (protocol n. 24045, 16 December 2024). Questionnaires were administered online with Qualtrics Online Surveys. Participation was voluntary, and participants were invited to complete the questionnaires in a laboratory setting. They were given 1 week to complete the survey, and data collection began immediately after receiving ethical approval and was completed within 1 month.

### 2.10 Statistical Analyses

#### 2.10.1 Descriptive Statistics and Reliability

We calculated the descriptives (mean, standard deviation, skewness and kurtosis) of psychological scales. Scales with skewnesses and kurtoses between −2 and 2 have a normal distribution of data. Reliabilities were calculated with Cronbach's alpha and McDonald's omega. Reliability coefficients higher than 0.70 indicate good reliability, between 0.60 and 0.70 are acceptable and between 0.50 and 0.60 are poor.

#### 2.10.2 Bivariate Correlations

We calculated bivariate correlations between psychological scales to analyse the presence or absence of relevant convergences and divergences between variables and also interconnections between variables measuring different psychological constructs.

#### 2.10.3 Confirmatory Factor Analysis (CFA)

We carried on different CFAs on scales measuring hope, mental health and psychological well‐being. Theses scale were used to estimate a general latent factor for each group of scales. Therefore, three general latent factors were estimated: a general factor for hope (hope) a general factor for mental health (mh) problems and a general factor for psychological well‐being (pwb). The factor scores or hope, mh and pwb were the used in mediation model analysis. Using factor scores derived from CFA allows for a more accurate representation of the latent constructs by accounting for measurement error and ensuring that the scores reflect the underlying theoretical dimensions, thereby improving the validity and reliability of subsequent analyses. The goodness‐of‐fit indexes were calculated to estimate the validity of CFA models. Models with *χ* ^2^/df ≤ 3, TLI ≥ 0.90, CFI ≥ 0.90, RMSEA ≤ 0.08, lower limit of 90% RMSEA ≤ 0.08, SRMR ≤ 0.08 are acceptable (Kline 2023). We used maximum likelihood as estimator because the observed variables were the global score of each subscales, with a wide range of values.

#### 2.10.4 Mediation Analysis

The mediation model predictor was the physical, social and psychological lockdown effects and the outcomes were pwb and mh. The mediators were hope and the emotion regulation components, CR and ES. CR and ES were standardised before performing the analysis. Significances of direct and indirect path coefficients were calculated and the difference between direct and indirect path coefficients was estimated, as well.

All statistical analyses were made with *Rstudio* using *lavaan* and *psych* packages.

## 3 Results

Table 1 shows the descriptives for each psychological scale. Except for few scales skewnesses and kurtoses are included between −2 and 2, indicating a good approximation to normal distribution in the collected data. Reliability coefficients (Cronbach's alpha and McDonalds' omega) are all sufficient, excepte for the Hope Herth Index‐Interconnectedness subscale, for which the reliability is poor but acceptable. Correlation with the Marlowe‐Crowne scale were significant for the Snyder Hope scale, Hope Herth Index, Emotion Regulation Questionnaire, Brief Symptom Inventory, Subjective Happiness Scale and Basic Psychological Needs Scale. In particular, correlations with the Marlowe‐Crowne are positive for hope scales (Snyder Hope scale‐Pathway and all Hope Herth Index subscales), psychological well‐being scales (Subjective Happiness Scale, Basic Psychological Needs Scale‐Autonomy, Competence and Relatedness) and with Emotion Regulation Questionnaire‐CR. Correlations were negative for mental health scales (Brief Symptom Inventory Depression and Anxiety). Although several correlations reached statistical significance, all coefficients were below |0.30|. Following widely accepted conventions for interpreting effect sizes (Funder and Ozer 2019), correlations of this magnitude are typically classified as ‘small’ and correspond to approximately 9% or less of shared variance (i.e., *r* ^2^ < 0.10). This does not rule out the possibility of response bias, but it suggests that social desirability shows only limited convergence with the psychological variables measured. Importantly, social desirability was used solely as a diagnostic indicator and was not statistically controlled for in subsequent analyses, as the observed associations did not indicate a level of overlap warranting covariate adjustment. Instead, it is reported here to allow readers to evaluate the potential impact of response bias on self‐reported measures.

**TABLE 1:** Descriptive statistics (mean, standard deviation, skewness, kurtosis), reliability coefficients (Cronbach's *α* and McDonald's *ω*) and bivariate correlations between psychological scales and measures of lockdown impact on life. Correlations with the social desirability scale (MC) are also reported.

Measures	Mean	SD	Skewness	Kurtosis	Cronbach's *α*	McDonald's *ω*	Correlation with MC	
Physical L.	2.65	0.77	0.17	1.00			−0.03	
Psychological L.	2.06	0.77	0.77	1.52			0.06	
Social L.	2.73	0.86	0.35	0.50			0.20	**
ERQ‐CR	30.53	5.34	−1.05	2.76	0.82	0.82	0.26	***
ERQ‐ES	14.23	5.15	0.12	−0.57	0.75	0.77	0.03	
SnHS‐pathways	11.73	1.71	−0.37	2.10	0.66	0.68	0.21	**
SnHS‐agency	11.83	1.89	−0.62	0.96	0.69	0.71	0.12	
HHI‐TF	11.59	1.98	−0.12	−0.04	0.67	0.70	0.24	**
HHI‐RE	12.53	1.77	−0.73	1.72	0.64	0.65	0.21	**
HHI‐I	11.76	2.02	−0.40	0.10	0.54	0.55	0.23	**
BSI18‐Som	4.64	4.71	1.29	1.38	0.81	0.82	−0.03	
BSI18‐Dep	6.82	5.10	0.88	0.08	0.83	0.85	−0.17	*
BIS18‐Anx	8.59	5.39	0.43	−0.56	0.86	0.87	−0.15	*
SHS	18.67	5.23	−0.49	0.09	0.84	0.84	0.16	*
BPNS‐Aut	26.21	4.69	−0.90	1.34	0.83	0.84	0.28	***
BPNS‐Com	20.50	3.90	−0.34	0.11	0.73	0.76	0.20	**
BPNS‐Rel	30.76	4.96	−0.56	0.15	0.78	0.79	0.25	***

Abbreviations: BPNS, Basic Psychological Needs Scale (Aut, autonomy; Com, competence; Rel, relatedness); BSI18, Brief Symptom Inventory‐18 items (Anx, anxiety; Dep, depression; Som, somatisation); ERQ, Emotion Regulation Questionnaire (CR, cognitive reappraisal; ES, expressive suppression); HHI, Hope Herth Index (I, interconnectedness; RE, readiness and expectancy; TF, temporality and future); L., lockdown; MC, Marlowe‐Crowne Scale of social desirability; SD, standard deviation; SHS, Subjective Happiness Scale; SnHS, Snyder Hope Scale. *p < 0.05, **p < 0.01, ***p < 0.001.

Table 2 also shows the bivariate correlations between the psychological scales. Not only are convergent scales correlated, but every scale is interconnected with scales measuring opposite or different constructs.

**TABLE 2:** Bivariate correlations between psychological scales.

Scales	1		2		3		4		5		6		7		8		9		10		11		12		13	
ERQ‐CR	—																									
2 ERQ‐ES	−0.11		—																							
3 SnHS‐pathways	0.41	***	−0.15	*	—																					
4 SnHS‐agency	0.33	***	−0.25	***	0.51	***	—																			
5 HHI‐TF	0.41	***	−0.27	***	0.52	***	0.63	***	—																	
6 HHI‐RE	0.44	***	−0.38	***	0.56	***	0.58	***	0.71	***	—															
7 HHI‐I	0.39	***	−0.45	***	0.43	***	0.48	***	0.55	***	0.69	***	—													
8 BSI18‐Som	−0.16	*	0.32	***	−0.21	**	−0.24	**	−0.30	***	−0.34	***	−0.32	***	—											
9 BSI18‐Dep	−0.33	***	0.40	***	−0.41	***	−0.47	***	−0.59	***	−0.58	***	−0.61	***	0.55	***	—									
10 BIS18‐Anx	−0.23	**	0.35	***	−0.36	***	−0.32	***	−0.43	***	−0.41	***	−0.39	***	0.71	***	0.70	***	—							
11 SHS	0.38	***	−0.35	***	0.51	***	0.44	***	0.64	***	0.59	***	0.60	***	−0.29	***	−0.60	***	−0.45	***	—					
12 BPNS‐Aut	0.26	***	−0.30	***	0.46	***	0.52	***	0.57	***	0.56	***	0.51	***	−0.30	***	−0.53	***	−0.41	***	0.60	***	—			
13 BPNS‐Com	0.25	***	−0.34	***	0.47	***	0.58	***	0.59	***	0.56	***	0.55	***	−0.33	***	−0.61	***	−0.47	***	0.53	***	0.58	***	—	
14 BPNS‐Rel	0.24	**	−0.36	***	0.37	***	0.30	***	0.46	***	0.52	***	0.55	***	−0.33	***	−0.50	***	−0.38	***	0.60	***	0.67	***	0.52	***

*Note*: *p < 0.05, **p < 0.01, ***p < 0.001.

Abbreviations: BPNS, Basic Psychological Needs Scale (Aut, autonomy; Com, competence; Rel, relatedness); BSI18, Brief Symptom Inventory‐18 items (Anx, anxiety; Dep, depression; Som, somatisation); ERQ, Emotion Regulation Questionnaire (CR, cognitive reappraisal; ES, expressive suppression); HHI, Hope Herth Index (I, interconnectedness; RE, readiness and expectancy; TF, temporality and future); SHS, Subjective Happiness Scale; SnHS, Snyder Hope Scale.

In relation to the three components of lockdown effects, bivariate correlations indicated that there were positive and significant correlations between the psychological and the physical impact of lockdown (*r* = 0.341, *p* < 0.001) and between the psychological and the social impact of lockdown (*r* = 0.263, *p* < 0.001). Therefore, a worsening of psychological condition resulted in a connection with the worsening of physical and social condition. Correlation between social and physical impact of lockdown was not significant (*r* = 0.121, *p* = 0.103).

**FIGURE 1:**
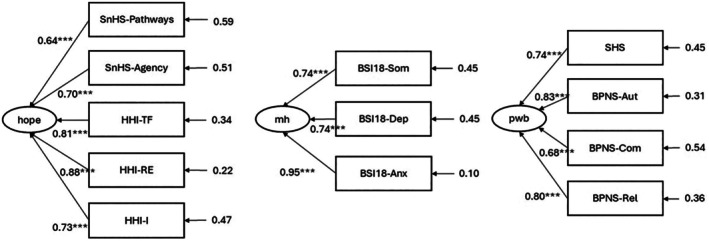
CFA models for hope, mental health (mh) and psychological well‐being (pwb). BPNS, Basic Psychological Needs Scale (Aut, autonomy; Com, competence; Rel, relatedness); BSI18, Brief Symptom Inventory‐18 items (Anx, anxiety; Dep, depression; Som, somatisation); HHI, Hope Herth Index (I, interconnectedness; RE, readiness and expectancy; TF, temporality and future); SnHS, Snyder Hope Scale; SHS, Subjective Happiness Scale.

Because of the significant convergence of scales measuring the same psychological construct, we decided to create CFA models in which each scale converged to a common general latent factor. Figure 1 shows the CFA models for estimating the general latent factor for hope, mental health, and psychological well‐being scales.

Table 3 reports the goodness‐of‐fit indexes of each CFA model. All values indicate a good fit for each model. Therefore, factor scores for the general factors hope, mental health, and psychological well‐being were estimated and used for mediation analysis.

**TABLE 3:** Goodness‐of‐fit indexes of CFA models for hope, mental health and psychological well‐being.

Model	*χ* ^2^	df	*χ* ^2^/df	TLI	CFI	RMSEA	90% RMSEA	SRMR
hope	443.06	10	44.31	0.950	0.975	0.109	0.051–0.171	0.028
mh	254.61	3	84.87	0.998	0.999	0.000	0.0–0.0	0.000
pwb	308.81	6	51.47	0.999	0.999	0.011	0.0–0.147	0.012

Abbreviations: CFI, Comparative Fit Index; mh, mental health; pwb, psychological well‐being; RMSEA, root mean square error of approximation; SRMR, standardised root mean square error; TLI, Tucker‐Lewis Index.

**FIGURE 2:**
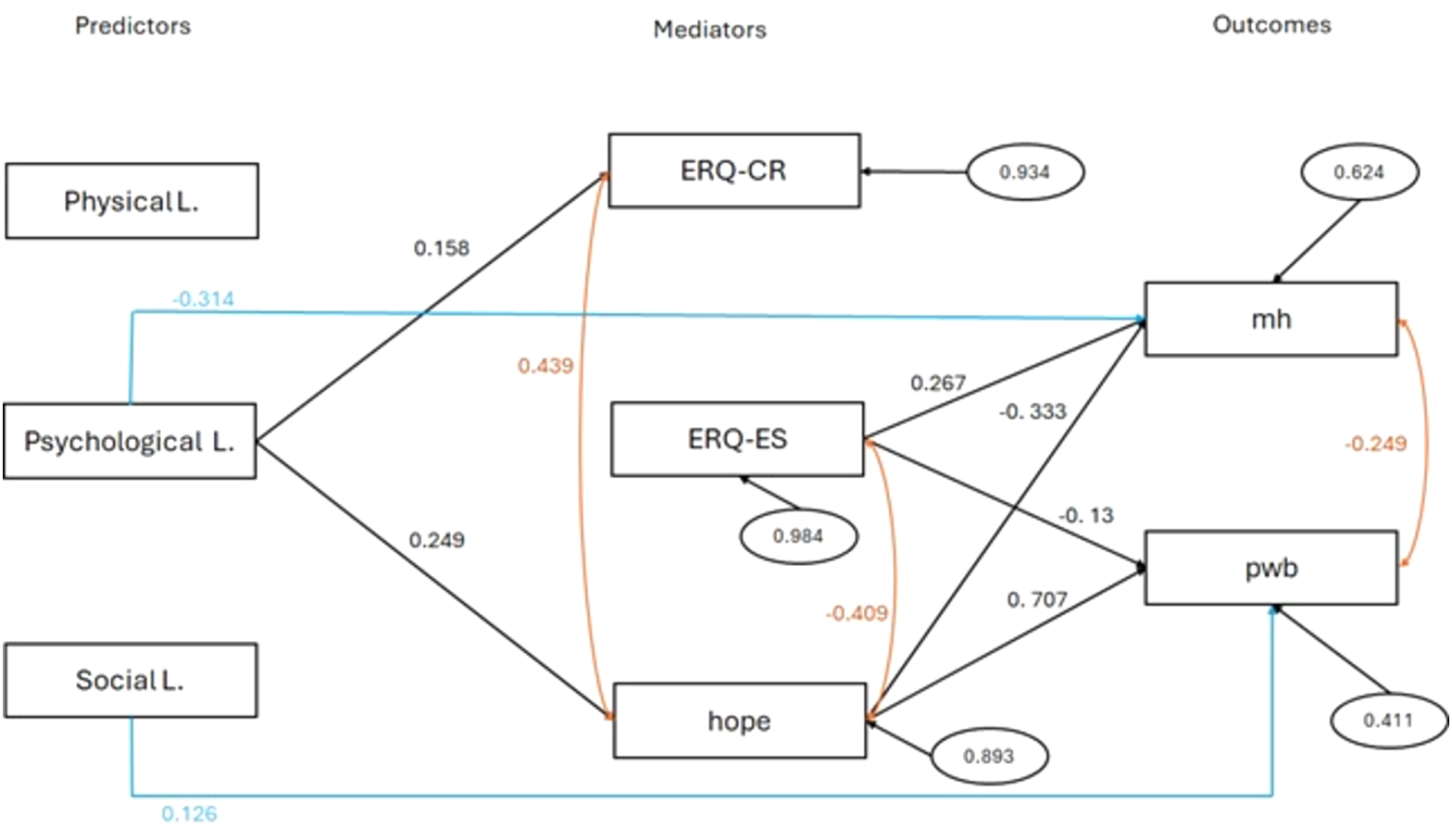
Model for mediation analysis. Only significant paths (*p* < 0.05) are reported with standardised coefficients (*β*). Blue lines indicate direct paths between predictors and outcomes. Red lines indicate correlations between variables. Standardised residuals are reported in ellipses. ERQ, Emotion Regulation Questionnaire (CR, cognitive reappraisal; ES, expressive suppression); hope, factor scores of hope scales; L., lockdown; mh, factor scores of mental health problems scales; pwb, factor scores of psychological well‐being scales.

Figure 2 shows the mediation model in which the three types of lockdown impacts (physical, psychological, and social impact).

Table 4 reports the direct and indirect path coefficients and relative *z* values and significances. In relation to direct effects, results showed that the psychological impact of lockdown affected mental health problems. The more negative the psychological impact, the higher the worsening of mental health. The social impact of lockdown was positively correlated with psychological well‐being, indicating that the improvement of social relationships after lockdown improved the psychological conditions of individuals.

**TABLE 4:** Path coefficients for raw (*b*) and standardised (*β*) scores.

Predictors		Mediators		Outcomes	*b*	*β*	SE	*z*	*p* (*z*)
Direct paths									
Physical L.		→		mh	0.262	0.078	0.211	1.244	0.214
Psychological L.		→		mh	−1.054	−0.314	0.225	−4.686	< 0.001
Social L.		→		mh	0.140	0.042	0.204	0.685	0.494
ERQ‐CR		→		mh	−0.040	−0.012	0.226	−0.178	0.859
ERQ‐ES		→		mh	0.897	0.267	0.217	4.138	< 0.001
Hope		→		mh	−1.085	−0.333	0.245	−4.435	< 0.001
Physical L.		→		pwb	−0.170	−0.047	0.183	−0.929	0.350
Psychological L.		→		pwb	0.055	0.015	0.195	0.283	0.777
Social L.		→		pwb	0.454	0.126	0.177	2.559	0.011
ERQ‐CR		→		pwb	−0.145	−0.040	0.197	−0.739	0.460
ERQ‐ES		→		pwb	−0.468	−0.130	0.189	−2.482	0.013
Hope		→		pwb	2.471	0.707	0.213	11.619	< 0.001
Physical L.		→		ERQ‐CR	0.096	0.096	0.076	1.260	0.208
Psychological L.		→		ERQ‐CR	0.158	0.158	0.078	2.022	0.043
Social L.		→		ERQ‐CR	0.102	0.102	0.074	1.379	0.168
Physical L.		→		ERQ‐ES	−0.121	−0.121	0.078	−1.550	0.121
Psychological L.		→		ERQ‐ES	0.096	0.096	0.080	1.199	0.230
Social L.		→		ERQ‐ES	−0.028	−0.028	0.076	−0.373	0.709
Physical L.		→		hope	0.127	0.124	0.076	1.666	0.096
Psychological L.		→		hope	0.257	0.249	0.079	3.268	0.001
Social L.		→		hope	0.043	0.042	0.074	0.583	0.560
Indirect paths									
Physical L.	→	ER_CR	→	mh	−0.004	−0.001	0.022	−0.176	0.860
Physical L.	→	ERQ‐ES	→	mh	−0.108	−0.032	0.075	−1.451	0.147
Physical L.	→	hope	→	mh	−0.138	−0.041	0.088	−1.560	0.119
Psychological L.	→	ER_CR	→	mh	−0.006	−0.002	0.036	−0.177	0.859
Psychological L.	→	ERQ‐ES	→	mh	0.086	0.026	0.075	1.152	0.249
Psychological L.	→	hope	→	mh	−0.278	−0.083	0.106	−2.631	0.009
Social L.	→	ER_CR	→	mh	−0.004	−0.001	0.023	−0.177	0.860
Social L.	→	ERQ‐ES	→	mh	−0.025	−0.008	0.068	−0.372	0.710
Social L.	→	hope	→	mh	−0.047	−0.014	0.081	−0.578	0.563
Physical L.	→	ER_CR	→	pwb	−0.014	−0.004	0.022	−0.637	0.524
Physical L.	→	ERQ‐ES	→	pwb	0.056	0.016	0.043	1.315	0.189
Physical L.	→	hope	→	pwb	0.314	0.087	0.191	1.649	0.099
Psychological L.	→	ER_CR	→	pwb	−0.023	−0.006	0.033	−0.694	0.488
Psychological L.	→	ERQ‐ES	→	pwb	−0.045	−0.012	0.042	−1.080	0.280
Psychological L.	→	hope	→	pwb	0.634	0.176	0.202	3.146	0.002
Social L.	→	ER_CR	→	pwb	−0.015	−0.004	0.023	−0.651	0.515
Social L.	→	ERQ‐ES	→	pwb	0.013	0.004	0.036	0.369	0.712
Social L.	→	hope	→	pwb	0.107	0.030	0.184	0.582	0.560
Direct–indirect difference									
Physical L.	→	hope	→	mh	−0.775	−0.231	0.262	−2.956	0.003
Psychological L.	→	hope	→	pwb	−0.579	−0.161	0.290	−1.996	0.046

*Note*: Significant coefficients (*p* < 0.05) of direct or indirect paths are in bold type.

Abbreviation: SE, standard error.

High levels of ES are connected to a higher level of mental health dysfunction and a lower psychological well‐being, while the CR component of emotion regulation did not show any significant effect on mental health and psychological well‐being. Hope showed a strong and positive effect on psychological well‐being and a negative effect on mental health.

In relation to indirect effect, only paths in which hope acted as mediator resulted significant if the predictor was the psychological impact of lockdown on mental health and psychological well‐being. The difference between the path coefficient of direct and indirect effect between psychological impact of lockdown and mental health problems resulted significant (*β* = −0.775, *p* < 0.01). Hope as mediator reduces the negative impact of lockdown on mental health. The difference between the path coefficient of direct and indirect effect between psychological impact of lockdown and psychological well‐being resulted also significant (*β* = −0.579, *p* < 0.05). Therefore, hope can improve the capacity of individuals to restore positive psychological condition after the lockdown.

## 4 Discussion

Our findings are consistent with previous studies and offer substantial support for hypothesis H1. A decline of physical, psychological and social conditions from the period immediately preceding the lockdown to its conclusion also exhibited connections with higher levels of somatization, anxiety, depression and lower levels of happiness and psychological well‐being. Notably, participants who experienced significant psychological distress following the lockdown reported greater psychological suffering.

Conversely, participants who reported stable or improved conditions appeared to demonstrate greater resilience, enabling them to cope with adverse circumstances and maintain a positive psychological state. These findings support earlier research, which demonstrated that individuals with higher resilience are better equipped to overcome generalised stress and adversity (Killgore et al. 2020; Tommasi et al. 2020).

Regarding emotion regulation, our findings further support the role of cognitive reappraisal in maintaining more stable psychological conditions. Cognitive reappraisal was found to be negatively correlated with measures of mental health impairment and positively correlated with measures of psychological well‐being, consistent with previous research (Peh et al. 2017; Vally and Ahmed 2020). Conversely, expressive suppression was positively correlated with measures of mental health impairment and negatively correlated with psychological well‐being, a result also supported by earlier studies (Dryman and Heimberg 2018; Peh et al. 2017).

Although prior literature has documented a potential mediating role for these two emotion regulation mechanisms—particularly cognitive reappraisal—in the relationship between adverse events and psychological outcomes (Graça and Brandão 2024; Laslo‐Roth et al. 2022), our mediation analysis found no significant mediating effects of either cognitive reappraisal or expressive suppression on the relationship between lockdown effects and mental health problems or psychological well‐being. Therefore, hypotheses H2 and H3 are not confirmed. The absence of mediation may reflect the context‐specific nature of emotion regulation during the lockdown, where external constraints limited the effectiveness of individual strategies. A plausible explanation may be that emotions are transient psychological states, and their impact is limited to a specific time frame. Future studies could include additional analyses (e.g., moderation or conditional process models) to explore the role of emotion regulation strategies in more depth.

In contexts of prolonged adversity, emotion regulation strategies, such as cognitive reappraisal and expressive suppression, may be insufficient. Reinterpreting a situation or suppressing negative emotions alone cannot effectively address challenges that cannot be resolved or controlled. In such circumstances, a different psychological resource may be required—one that enables individuals to endure until the adversity subsides. Although previous research has documented significant mediating effects of cognitive reappraisal and expressive suppression on psychological outcomes (e.g., Peh et al. 2017; Vally and Ahmed 2020; Laslo‐Roth et al. 2022), our results did not replicate these effects. This divergence may be attributable to methodological limitations in earlier studies, which often failed to account for the context‐dependent nature of emotion regulation under conditions of prolonged adversity, such as pandemic‐related lockdowns. Moreover, prior models have generally conceptualised emotion regulation as a stable mediating construct, without considering the temporal variability of emotional states or the external constraints that may attenuate the efficacy of individual regulatory strategies. The findings of the present study suggest that emotion regulation mechanisms may be insufficient to mediate psychological outcomes in contexts characterised by sustained stress and limited controllability.

From this perspective, hope emerges as a critical psychological resource, empowering people to cope with extended periods of stress. Previous studies have highlighted the fundamental role of hope in maintaining psychological well‐being for individuals navigating difficult circumstances (Richards 2002). Hope not only helps individuals manage long‐term stress but also supports their ability to overcome persistent challenges.

Our mediation analysis revealed that hope plays a significant role in buffering the negative effects of lockdown on mental health and psychological well‐being, confirming H4. Specifically, hope appears to attenuate the adverse psychological impact of lockdown and to enhance individuals' capacity to respond adaptively to stress, thereby preserving their psychological well‐being despite widespread disruptions to daily life. Notably, we observed a full mediation effect, where the psychological impact of lockdown on well‐being was entirely mediated by hope, indicating that the direct effect of lockdown on well‐being was negligible.

These findings highlight hope as an important factor for fostering resilience in the face of significant adversities. As demonstrated in prior research, psychological support, training or therapy can cultivate a strong sense of hope, equipping individuals to cope more effectively with negative events (Chan et al. 2019; Reis and Hoppe 2015). Therefore, psychological interventions aimed at enhancing hope may serve as an effective strategy to strengthen resilience and preserve quality of life.

The contribution of the present research lies in emphasising the potential usefulness of targeting hope within psychological interventions aimed at promoting resilience and preserving quality of life.

While previous studies (Chan et al. 2019; Reis and Hoppe 2015) have shown that hope can be cultivated through psychological support and training, this research contributes by positioning hope not merely as a desirable outcome, but as an active and central component of intervention strategies. Specifically, it suggests that psychological programmes focused on enhancing hope may serve as an effective and preventive approach to strengthen individuals' resilience in the face of adversity, with positive implications for overall well‐being.

Our study has several limitations. First, the participant sample consisted of a convenience group of university students, which may limit the generalizability of our findings. Additionally, more than 80% of the participants were female, introducing a potential gender bias. Although this study focused on university students, the psychological mechanisms investigated—such as emotion regulation, hope, and well‐being—are not exclusive to this population. These constructs are broadly relevant across different age groups and cultural contexts. Therefore, future research could extend the current model to non‐student samples and cross‐cultural settings, encompassing individuals from different professional backgrounds, age groups and life circumstances to examine the generalizability and potential contextual variations of the observed relationships.

Moreover, our study did not account for material resources or benefits (e.g., socioeconomic status, income level, living conditions, access to remote work, availability of internet connections) that might have influenced participants' ability to cope with the lockdown. Although our primary focus was on their social and psychological conditions, considering these factors could provide a more comprehensive understanding of how individuals navigate such challenging periods.

In conclusion, our study showed that hope had a significant mediating role between lockdown effect on individual life and mental health and psychological well‐being. Emotion regulation through cognitive reappraisal or expressive suppression showed no significant mediating influence. Therefore, hope represents the human psychological resource that best helps human beings to deal with prolonged and general adversities.

## Author Contributions

**Marco Tommasi:** conceptualization, formal analysis, investigation, writing – original draft, writing – review and editing, methodology. **Simone Arnò:** software, data curation, review and editing. **Aristide Saggino:** supervision, writing – review and editing, validation.

## Funding

The authors have nothing to report.

## Ethics Statement

Privacy of participants was guaranteed according to the Italian and European law (Italian law no. 196/2003 and EU GDPR 679/2016, respectively). This study followed the 1964 Declaration of Helsinki ethical principles for research involving human subjects and was approved by the Institutional Review Board of Psychology of the Department of Psychology (protocol no. 24045; 16 December 2024).

## Consent

Informed consent was obtained from all individual adult participants included in the study.

## Conflicts of Interest

The authors declare no conflicts of interest.

## Data Availability

The data that support the findings of this study are openly available in resilience at https://osf.io/qakme/files/osfstorage.
